# Null tDCS Effects in a Sustained Attention Task: The Modulating Role of Learning

**DOI:** 10.3389/fpsyg.2018.00476

**Published:** 2018-04-06

**Authors:** Noa Jacoby, Michal Lavidor

**Affiliations:** Cognitive Neuroscience Laboratory, Department of Psychology, Gonda Brain Science Center, Bar-Ilan University, Ramat Gan, Israel

**Keywords:** tDCS, ADHD, sustained-attention, learning-effect, CPT, DLPFC, cerebellum

## Abstract

The purpose of this study was to investigate sustained attention through modulation of the fronto-cerebral network with transcranial direct current stimulation (tDCS) in adults with attention-deficit/hyperactivity disorder (ADHD) and control participants. Thirty-seven participants (21 with ADHD) underwent three separate sessions (baseline, active tDCS, and sham) and performed the MOXO Continuous Performance Test (CPT). We applied double anodal stimulation of 1.8 mA tDCS for 20 min over the left and right dorsolateral prefrontal cortex (DLPFC), with the cathode over the cerebellum. Baseline session revealed significant differences between ADHD and control participants in the MOXO-CPT attention and hyperactivity scores, validating the MOXO as a diagnostic tool. However, there were no tDCS effects in most MOXO-CPT measures, except hyperactivity, due to a significant learning effect. We conclude that learning and repetition effects in cognitive tasks need to be considered when designing within-subjects tDCS experiments, as there are natural improvements between sessions that conceal potential stimulation effects.

## Introduction

Attention-deficit/hyperactivity disorder (ADHD) is a neuropsychiatric disorder which is typified by developmentally inappropriate symptoms of inattention, impulsivity, and hyperactivity, that are present in various surroundings (e.g., school and home), leading to deficits in social, educational or work settings, and impacts different domains of cognitive skills such as executive functions (EF) and cognitive control (Lijffijt et al., 2005; Barkley, 2010; American Psychiatric Association [APA], 2013). The estimated prevalence is between 5 and 8% in children, and for up to 60% of the children with ADHD, the disorder continues into adulthood with a prevalence of 2.5–4% in adults (Mannuzza et al., 2003; Kooij et al., 2005; Fayyad et al., 2007).

Adults with ADHD usually experience problems engaging in everyday activities (Barkley, 2011b; Ek and Isaksson, 2013), and they are at a greater risk for lower socioeconomic and professional status, poorer academic achievements, antisocial behavior, and various parenting and relationship difficulties (Barkley, 2002; Adler et al., 2008; Johnston et al., 2012; Shaw et al., 2012).

Attention-deficit/hyperactivity disorder arises from disorders in the prefrontal cortex (PFC) which is also the main region regulating EF and self-monitoring (Barkley, 1997; Brown, 2013). EF is an umbrella term comprising a wide range of complex cognitive processes that enable goal-directed behavior, such as planning and executing tasks while staying concentrate and focused, dysfunction in such processes has been long recognized as the hallmark of ADHD (Barkley, 1997, 2012; Castellanos et al., 2006). A large body of neuroimaging studies confirm the role of the PFC, and its connectivity to other regions, as the main region regulating the EF (Miyake et al., 2000; Elliott, 2003; Alvarez and Emory, 2006). Furthermore, ADHD patients demonstrate patterns of hypoactivation in brain regions responsible for EF; one of the most evidence-based models is the prefrontal–striatal circuit that was expanded to include cerebellar involvement (Krain and Castellanos, 2006; Castellanos and Proal, 2012; Cortese et al., 2012). In a functional magnetic resonance imaging (fMRI) study, Schneider et al. (2010) compared adults with ADHD and control participants while they performed continuous performance test (CPT), their findings demonstrated impaired activation of the PFC, cerebellum, cingulate cortex (ACC), the fronto-striatal and parietal attentional network in ADHD subjects.

Pharmacologic treatments with stimulants (such as methylphenidate and amphetamines) for ADHD patients have been reported to be highly effective (Feldman and Reiff, 2014; Craig et al., 2015). However, about 30–40% of patients do not respond well to pharmacological treatment; they experience side effects or continue to have unresolved significant impairments that lead to treatment discontinuation (Castells et al., 2013; Clavenna and Bonati, 2014; Childress and Sallee, 2014). Therefore, there is a need to develop new non-pharmacological alternatives for patients with ADHD.

Transcranial direct current stimulation (tDCS) is already in use as an alternative treatment for neuropsychiatric disorders (Kuo et al., 2014). tDCS induces short-term changes in cortical excitability by employing weak electrical currents over the scalp (Nitsche and Paulus, 2000). Anodal and cathodal stimulation can induce enhancement or reduction in neuronal activity, respectively, and influence brain function (Nitsche and Paulus, 2000). The physiological effects of tDCS last about 1 h subsequent to several minutes of stimulation or longer after multiple stimulation sessions (Nitsche and Paulus, 2000; Nitsche et al., 2003; Snowball et al., 2013).

The exploration of attentional processes in ADHD patients via tDCS is relatively new, and different studies showed a diversity and inconsistent findings (Coffman et al., 2014; Reteig et al., 2017). tDCS attention studies applied stimulation over areas involved primarily in top-down processing. In a sustained attention task, Nelson et al. (2014) revealed that anodal tDCS over the left DLPFC had a positive effect on vigilance decrement in a simulated air traffic controller task. Mannarelli et al. (2016) used a P300 novelty task to explore the role of the cerebellum in attentional tasks and found that cathodal cerebellar tDCS significantly decreased N1, N2, and P3 components for both the target and novel stimuli. The authors assumed that cathodal cerebellar stimulation affected frontal and parietal regions by decreasing their inhibitory control, which led to increased activation in those regions and affected their functional synchronization. Therefore, the study indicates that the cerebellum might regulate the activation and inhibition levels in attentional networks (Mannarelli et al., 2016).

In a recent fMRI study, Sotnikova et al. (2017) demonstrated the spreading effect of tDCS in a sample of adolescents ADHD patients. They applied left DLPFC anodal stimulation while performing a working memory task combined with Go/NoGo task. In addition to improvement in the task performances, tDCS led to increased neuronal activation and connectivity, that was not limited to the left DLPFC, but also in more remote brain regions (e.g., working memory and executive control networks). Moreover, Soff et al. (2017) used a version of a CPT test and were able to demonstrate that anodal left DLPFC tDCS applied over five consecutive days in adolescents with ADHD led to a significant reduction of ADHD symptoms, primarily of inattention and hyperactivity, which lasted for 1 week after the end of stimulation, demonstrating a long-lasting effect of tDCS. Bandeira et al. (2016) tested children with ADHD and applied anodal tDCS over the left DLPFC. This montage generated improved performance on all measures – accuracy during a sustained attention task, signal detection, switching ability, error rate and run time. When bilateral stimulation was applied to the DLPFC while adolescent ADHD patients performed sustained attention task (Go/NoGo), Soltaninejad et al. (2015) found that cathodal stimulation to the left DLPFC improved no-go accuracy (compared to sham), while anodal left DLPFC increased the proportion of correct responses (go accuracy), compared to sham stimulation.

Although tDCS studies have shown enhancement of attentional processes and EFs such as response inhibition, task switching, attention and working memory (Jacobson et al., 2012; Reteig et al., 2017), there is still lack of information and diversity in the results regarding tDCS influence on adult ADHD patients and the comparison to healthy adults. Therefore, in the current work, we addressed the prefrontal-cerebral network and applied double anodal tDCS, including the right and left DLPFC, with the cathodal on the cerebellum, a montage that to our knowledge was not applied before.

Our hypotheses were:

(1) We expected that baseline measures and self-reported symptoms will be impaired in the ADHD group when compared to healthy participants.
(2) We hypothesized that anodal prefrontal tDCS would result in improved performance of the attention and EF tasks compared to baseline and sham conditions. We had no consistent prior background as to whether such predicted improvements would interact with subject groups, that is ADHD and control participants.

## Materials and Methods

### Overview

The core deficit in individuals with ADHD is the control over behavioral inhibition and self-regulation (Barkley, 1997). It has been suggested that PFC dysfunction and cerebral involvement are the main cause of this deficit. According to this concept, characteristic deficits of inattention, hyperactivity and impulsivity are identified with EF deficits and the PFC plays a crucial role in its regulation. Therefore, a CPT test was chosen to assess the participants performances. In addition, we used two self-report scales: adult ADHD self-report scale (ASRS; Kessler et al., 2005), which contains five factorial subscales, and the Barkley deficits in executive functioning scale (BDEFS; Barkley, 2011a) with eight factorial subscales.

### Materials

#### Participants

Thirty-seven participants between the ages 19–29 (*M* = 23.03, *SD* = 2.54;21 ADHD, 12 females; 16 controls, 7 females) were recruited from the students community of Bar-Ilan University. They were all right-handed and had normal or corrected-to-normal visual acuity, without a present or past history of neurological or psychiatric disorders (except ADHD) and were not on chronic medications (other than stimulant medications for ADHD). All the participants were asked to complete the ASRS and BDEFS questioners prior to their arrival. ADHD subjects were asked to produce a formal diagnosis (by a neurologist specialist or psychiatrist). In addition, they were all above the median in at least 10 out of 13 scales and subscales of the questionnaires.

Three subjects who scored 3.3 – 1.5 standard deviations higher than the control group in the main scales of the ASRS and BDEFS, in addition to scoring above the median in 10 out of 13 scales and subscales, were included in the ADHD group, although they did not produce a formal diagnosis. All the subjects were instructed to get at least 8 h of sleep prior to the experimental session, to avoid caffeine or nicotine for at least 2 h before the experimental session, and ADHD subjects were instructed to avoid any kind of medications for ADHD on the day of the experimental meeting. Informed consent was obtained from all participants, they were awarded either with course credit or a financial compensation (200 NIS; equivalent to about 50$) for their participation. The study was accepted by the Bar-Ilan ethics committee and was conducted in agreement with the Declaration of Helsinki guidelines.

#### tDCS Protocol

Double anodal bilateral tDCS was delivered by a constant current stimulator (Neuroconn, Ilmenau, Germany) with a direct current of 1.8 mA via three saline-soaked surface sponge rubber electrodes (anode 3 cm × 3 cm; cathode 5 cm × 7 cm). The active electrodes were placed over F4 and F3, a location atop the right and left DLPFC, according to the international 10–20 system for EEG electrode placement (Sela et al., 2012); the cathode electrode was centered over the cerebellar cortex, 1 cm below the inion (corresponding approximately to the projection of cerebellar lobule VII onto the scalp; Mannarelli et al., 2016). The placement of the cathodal electrode was not lateralized since we wanted to take advantage of the tDCS spreading effect and to gain bilateral effect on the region (Stagg et al., 2013; Sotnikova et al., 2017). The stimulation was applied for 20 min with a 30 s ramp (including fading out). For the sham condition, the electrodes were placed identically to the active stimulation, but the stimulator was turned off automatically after 30 s with a 30 s ramp, to generate the same sensation as active condition (Weiss and Lavidor, 2012). After 20 min of stimulation ended, the participants performed the MOXO-CPT, the procedure was the same for the sham condition; participants started the MOXO-CPT after 20 min. All participants were blind to the type of tDCS delivered in each session. To assess individual subjective effects of tDCS, at the beginning and at the end of each tDCS session participants were asked to fill out an affect questionnaire “Positive and Negative Affect Schedule” (PANAS; Watson et al., 1988).

#### MOXO-CPT

MOXO CPT (Berger and Goldzweig, 2010) is a standardized computerized test designed to diagnose ADHD related symptoms. MOXO-CPT is an 18.2-min test that includes visual and auditory distractor stimuli, it is composed of eight blocks (59 trials each) of 34 targets and 25 non-target stimuli. In each trial, a card was presented as an attended target, and participants’ task was to respond only to a certain card. The target was presented for 500, 1,000, or 4,000 ms, followed by a “void” period of a similar duration. The stimulus remained on the screen for the full duration irrespective of response.

**FIGURE 1:**
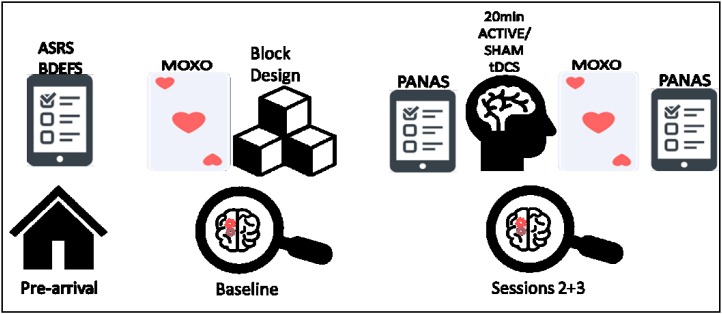
From left to right, schematic illustration of the experiment procedure; including the prior questionnaires, baseline measurements (MOXO-CPT and Wechsler block design), and the active/sham tDCS sessions (included PANAS questioner and MOXO-CPT).

Participants were instructed to respond as quickly as possible to target stimuli presented on screen by pressing the spacebar once, and only once. Participants were instructed to avoid responding to any other stimuli except for the exact target card and to use only the spacebar. Eight blocks which comprise three types of distractions were presented: (a) pure visual distractors (blocks 2 and 3; e.g., animated barking dog); (b) pure auditory distractors (blocks 4 and 5; e.g., barking sound); and (c) a combination of both (blocks 6 and 7; e.g., animated barking dog with the sound of barking). Visual distractors were presented at one of four spatial locations on the screen, including down, up, left, or right. The distractions had two load levels; in the 2nd, 4th, and 6th blocks, only one distractor was presented at a time, while in the 3rd, 5th, and 7th blocks two distractors were presented simultaneously. Distractor onset could be presented during the void period as well, hence was not synchronized with target onset. Distractors were presented for 8 s, with a fixed interval of a 0.5 s between two distractors. An illustration of the task is presented in **Figure 1**.

The MOXO-CPT assesses attention along four criteria, (a) Attention: number of correct responses to target not bound by any time frame (with a maximum of 272 correct responses). (b) Timing: number of correct responses only while the target is on screen. (c) Impulsivity: number of impulsive commissions performed in initial response to a non-target stimulus. (d) Hyperactivity, remaining commission errors not counted as impulsivity, for example, multiple spacebar presses (as opposed to initial) or random key pressing.

#### Wechsler Block Design

For controlling possible IQ differences between the groups, the Wechsler block design subtest was administrated. This subtest is relatively short and apparently has the highest correlation with the Wechsler’s general IQ score- G (Maxwell, 1959). Standardized scores were used as an IQ indicator.

#### Positive and Negative Affect Schedule (PANAS)

The PANAS (Watson et al., 1988) questionnaire collects subjective estimates of affect that can influence performances, therefore the questionnaire serves to assess correlations between individual subjective affect and objective effects of tDCS on tasks performances.

#### The Barkley Deficits in Executive Functioning Scale (BDEFS)

The BDEFS (Barkley, 2011a) has 89 items where behavior is self-rated on a Likert scale (1 = rarely or not at all, 4 = very often). The BDEFS has five-factor scores: self-management of time, self-organization, self-restraint, self-motivation, and self-regulation of emotions. Other measures can be calculated such as a total EF summary score, a significant symptoms score, and an ADHD-EF Index.

The BDEFS translation to Hebrew. In order to translate the questionnaire in the most reliable way, we took the following steps: first, we translated it to Hebrew, next, a research assistant, which was not previously exposed to the questionnaire, translated it back to English, and we compared and corrected the translation. Finally, two different researchers, both English and Hebrew native speakers, examined the translation and the original questionnaire and their comments were corrected. In the current study, Cronbach’s alpha reliability for the total scale (89 items) was 0.98.

#### Adult ADHD Self-Report Scale (ASRS)

The ASRS is an 18 items scale (Kessler et al., 2005) that measures the frequency of symptoms, i.e., how often ADHD symptoms occur, and it is directly based on the ADHD criteria in the DSM-IV-TR (American Psychiatric Association [APA], 2000). The ASRS has a two-factorial structure with an inattention scale and a hyperactivity/impulsivity scale. Additionally, ASRS total summary score, screener score, and clinically significant symptoms score are given. In the current study, Cronbach’s alpha reliability for the ASRS total scale (18 items) was 0.93.

### Procedure

**FIGURE 2:**
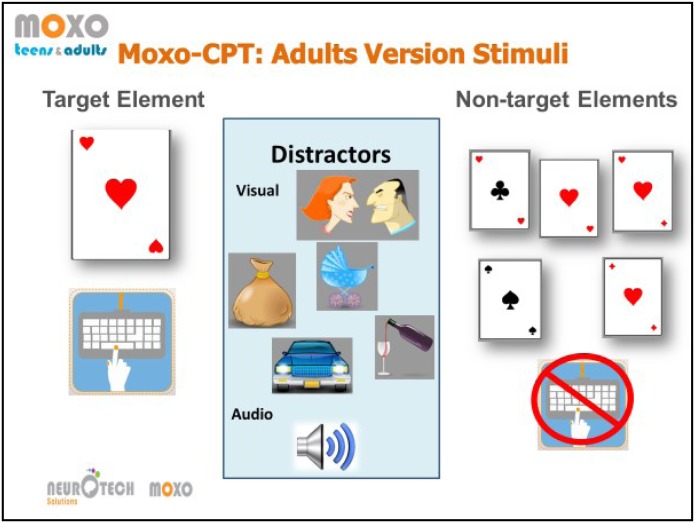
Illustration of the stimui in the MOXO-CPT task. From left to right, the target card and the adequate response, example of the visual distractors with the corresponding auditory stimului, and the non-target stimuli cards. The illustration was provided by Neurotech Solutions Ltd.

This study utilized a within-subject, cross-over design consisting of three phases with a 1-week interval between sessions: baseline (without tDCS intervention), active tDCS and sham stimulation. The baseline session was always the first session, the tDCS sessions were counterbalanced across all the participants. Since this was the first time we use the MOXO-CPT in our laboratory, baseline measurements were taken in order to examine the level of distinction between ADHD and control participants at the first time they are being exposed to the test. In addition, it was used to evaluate learning effect. Prior to their arrival, participants filled the ASRS, BDEFS and demographic questionnaires, at their arrival, participants signed the consent form and started the baseline session which included MOXO-CPT and Wechsler block design. In the stimulation sessions, the participant filled the PANAS questionnaire at their arrival, after 20 min of tDCS they performed the MOXO-CPT, and then filled the PANAS once again before they left. See **Figure 2** for a schematic illustration of the procedure.

### Data Analysis

All subjects tolerated the tDCS well and no adverse effects were reported. From the original sample, one control participant dropped out and did not complete the experimental sessions. In addition, one ADHD participant was excluded from the analysis, since his reaction times and error rates were more than 3.5 standard deviations above the mean of the respective group. Eventually, the analysis was conducted with a sample of 35 participants; 20 ADHD and 15 control participants. All data were analyzed using SPSS (version 21.0), MANOVA analysis was conducted to examine baseline differences between ADHD and controls in MOXO-CPT standard measures (attention score, hyperactivity, impulsiveness, and timing), reaction times were analyzed separately. For tDCS effects repeated measures analyses of variance (ANOVA) were conducted with the factors session type (baseline/tDCS active/tDCS sham) and group (ADHD/control) for all MOXO-CPT dependent measures.

## Results

### Baseline Assessments

There were no significant differences between the groups (ADHD/control) in all the demographic measures. Specifically, age [*t*_(33)_ = 0.81, *p* > 0.05], gender distribution (chi square = 0.24, *p* = 0.625), education years [*t*(33) = -1.66, *p* > 0.05] and Wechsler block design *Z* score [*t*_(33)_ = -0.23, *p* > 0.05] did not differ between the two groups. The gender distribution and average background variables are presented in **Table 1**.

**Table 1:** Means and standard deviations of demographic and MOXO-CPT baseline measures for ADHD and control participants.

		ADHD (*N* = 20)		Control (*N* = 15)	
			
		*M*	*SD*	*M*	*SD*
	Age	22.75	2.80	23.47	2.29
Demographic	Education years	13.45	1.23	13.07	0.26
	Gender	9 (males)		8 (males)	
	Block design	12.40	2.74	12.20	2.21
	Attention score^∗^	265.45	0.86	269.00	0.99
	Hyperactivity score^∗^	7.90	6.68	2.13	1.69
	Impulsivity score	15.95	3.21	7.07	3.71
MOXO-CPT	Timing score	206.75	6.42	216.13	7.41
Measures	Attention RT	561.80	47.27	542.79	51.50
	One distractor RT	572.73	48.49	552.99	54.46
	Two distractors RT	575.79	52.30	558.66	59.49
	Timing RT	538.78	45.18	523.16	42.31


**^∗^p < 0.05.**

In order to assess baseline performances differences between participants with ADHD and controls using the MOXO-CPT standard measures, a one-way MANOVA was conducted. The dependent variables were the score of attention, timing, impulsiveness and hyperactivity. The full model was significant as assessed with Wilke’s lambda [Wilk’s Λ = 0.71, *F*_(4,30)_ = 3.13, *p* = 0.029, $\eta_{p}^{2}$ = 0.29]. Univariate analyses indicated that the groups differed on attention [*F*_(1,33)_ = 7.35, *p* = 0.011, $\eta_{p}^{2}$ = 0.18] and hyperactivity scores [*F*_(1,33)_ = 10.01, *p* = 0.003, $\eta_{p}^{2}$ = 0.23], but not on impulsivity [*F*_(1,33)_ = 3.26, *p* = 0.08, $\eta_{p}^{2}$ = 0.09] and timing scores [*F*_(1,33)_ = 0.92, *p* > 0.1, $\eta_{p}^{2}$ = 0.03]. These findings indicate that participants with ADHD had more hyperactivity mistakes and had higher rates of target misses compared to control participants. Means and standard deviations for these measures are presented in **Table 1**.

Furthermore, a one-way MANOVA was conducted in order to examine reactions time differences between ADHD and controls participants in MOXO-CPT, baseline session. The dependent variables were attention RTs (reaction time) of correct responses, timing RTs (RTs of correct responses only while targets are displayed), RTs for one and two distractors. The full model was not significant as assessed with Wilke’s lambda [Wilk’s Λ = 0.91, *F*_(4,30)_ = 0.69, *p* > 0.1, $\eta_{p}^{2}$ = 0.08].

### tDCS Analyses

The PANAS affect questionnaire did not differ significantly between tDCS conditions, as paired samples *t*-test indicates; *t*_(34)_ = 1.51, *p* = 0.39. Mixed design Repeated measures ANOVAs were used to examine the effects of tDCS on MOXO-CPT performances, with the factors session type (baseline/tDCS active/tDCS sham) and group (ADHD/control) for MOXO-CPT attention score, hyperactivity score, impulsivity score timing score, attention RT and timing RT. Means and standard deviations of MOXO-CPT for session type by ADHD and control participants are presented in **Table 2**.

**Table 2:** Means and standard deviations of the MOXO-CPT measures as a function of stimulation condition and group (with or without ADHD).

Stimulation condition		Attention RT^∗^		Timing score^∗^		1 Distractor^∗^		2 Distractors^∗^		Hyperactivity score^∗^	
						
		*M*	*SD*	*M*	*SD*	*M*	*SD*	*M*	*SD*	*M*	*SD*
Baseline	ADHD	561.80	47.25	206.75	25.65	572.73	48.49	575.79	52.31	7.90	6.68
	Control	542.79	51.50	216.13	32.43	552.99	54.46	558.66	59.50	2.13	1.68
	Total	553.65	49.30	210.77	28.68	564.27	51.32	568.45	55.32	5.43	5.87
Sham	ADHD	546.96	53.48	220.00	26.62	558.94	52.08	568.87	53.54	7.00	6.33
	Control	517.30	44.97	234.47	24.56	527.13	48.69	534.96	50.49	2.73	3.79
	Total	534.25	51.51	226.2	26.40	545.31	52.41	554.34	54.24	5.17	5.74
Active	ADHD	541.51	50.83	220.60	24.90	552.19	53.94	556.49	54.44	4.40	4.43
	Control	520.46	49.23	230.07	25.36	532.46	52.66	542.21	54.53	2.60	4.29
	Total	532.46	50.53	224.66	25.18	543.73	53.53	550.37	54.15	3.63	4.40


**^∗^p < 0.05, a significant main effect of stimulation condition due to learning effect, as both sham and active stimulation significantly differed from baseline*.*

For attention RT, there was a significant main effect of session type [*F*_(2,66)_ = 9.02, *p* < 0.001, $\eta_{p}^{2}$ = 0.22]. A *post hoc* multiple comparisons Bonferroni test showed a significant difference in baseline (*M* = 552.29) and sham tDCS (*M* = 532.19; *p* = 0.003) and a significant difference between baseline and active tDCS (*M* = 530.99; *p* = 0.004). These results indicate a learning effect. There was no interaction effect of group (ADHD/control) and session type [*F*_(2,66)_ = 0.50, *p* = 0.61]. Repeated measures ANOVA with a Greenhouse–Geisser correction for attention score showed no significant main effect of session type [*F*_(1.6,52.93)_ = 0.10, *p* = 0.86] and no interaction effect [*F*_(1.6,52.93)_ = 2.57, *p* = 0.09].

For timing score, there was a significant main effect of session type [*F*_(2,66)_ = 15.24, *p* < 0.001, $\eta_{p}^{2}$ = 0.32]. A multiple comparisons Bonferroni test showed a significant difference between the performance in baseline (*M* = 211.44) and sham tDCS (*M* = 227.23; *p* = 0.002) and a significant difference between baseline and active tDCS (*M* = 225.33; *p* = 0.009), this effect also indicate a learning but not stimulation effect.

For one distractor RT there was a significant main effect of session type [*F*_(2,66)_ = 8.02, *p* = 0.001, $\eta_{p}^{2}$ = 0.20]. A multiple comparisons Bonferroni test showed a significant difference between the performance in baseline (*M* = 562.86) and sham tDCS (*M* = 543.03; *p* = 0.002) and a significant difference between baseline and active tDCS (*M* = 542.32; *p* = 0.009), this effect also indicates a learning effect. For two distractors RT, there was a significant main effect of session type [*F*_(2,66)_ = 5.32, *p* = 0.007, $\eta_{p}^{2}$ = 0.14]. A multiple comparisons Bonferroni test showed a significant difference between the performance in baseline (*M* = 567.23) and sham tDCS (*M* = 551.03; *p* = 0.03) and a significant difference between baseline and active tDCS (*M* = 549.35; *p* = 0.033), this effect also indicates a learning effect.

Regarding impulsivity, there were no significant main effect of session type [*F*_(2,66)_ = 0.28, *p* = 0.76], and no interaction effect [*F*_(2,66)_ = 1.19, *p* = 0.31].

The only measure that showed specific tDCS effects was the hyperactivity score (when the analysis used a Greenhouse–Geisser correction). There was no main effect of session type [*F*_(1.65,54.52)_ = 2.84, *p* = 0.077, $\eta_{p}^{2}$ = 0.08]. However, the analysis showed a significant main effect for ADHD [*F*_(1,33)_ = 6.86, *p* = 0.013, $\eta_{p}^{2}$ = 0.17], so the ADHD group (*M* = 6.43) had higher hyperactivity score then the control group (*M* = 2.48), and there was a significant interaction effect [*F*_(1.65,54.52)_ = 4.07, *p* = 0.029, $\eta_{p}^{2}$ = 0.11]. A multiple comparisons *post hoc* Bonferroni test showed a significant difference between the performance in sham (*M* = 5.17, *SD* = 5.74) and active tDCS (*M* = 3.63, *SD* = 4.43; *p* = 0.037), baseline measure did not significantly differ from the other measures (*M* = 5.4, *SD* = 5.87). Paired-sample *t*-test (with α correction of 0.02) showed that the difference between active to sham tDCS is significant only for ADHD [*t*_(19)_ = 3.2, *p* = 0.005] participant and not for controls [*t*_(14)_ = 0.27, *p* = 0.72].

## Discussion

Our primary question was whether tDCS would differently influence adults with ADHD compared to control participants. More specifically, we hypothesized that unlike baseline and sham measures, active anodal stimulation of the left and right DLPFC with cathodal on the cerebellum would result in improved performances of the MOXO-CPT and this improvement would be greater for ADHD participants in comparison to controls. Furthermore, we hypothesized that the MOXO-CPT baseline measurement would distinguish between ADHD and control participants.

Regarding our first hypothesis, it was not confirmed since we were not able to demonstrate improved performances in MOXO-CPT measures of attention, timing, impulsivity, attention RT and both of distractors load levels RT following stimulation. A possible explanation for the lack of tDCS effects is due to significant learning effects in attention score, timing score, attention RT, and distractors RT. For all these measures, although the main effect for session type (baseline/active/sham) was significant, *post hoc* analysis revealed that the significant differences were between baseline measures to sham stimulation, and between baseline sessions and active tDCS, there was no significant difference between sham and active stimulation for these measures. Furthermore, due to the major learning effect, it was not possible to differentiate between ADHD and control participants from the second time the test was applied. Due to the observed learning effects, results of test re-test were not sensitive enough to detect possible tDCS enhancing effects.

A possible cause for the observed learning effect in the MOXO-CPT, that potentially washed-out stimulation effects, is the lack of randomization, as blocks and trials order in each block is always identical. Perhaps if the blocks would have been presented in a random order, and the sequences of the trials in each block were randomized as well, it might have reduced learning effects. That could make the MOXO-CPT more suitable for test–retest and repeated measures design.

Referring to our second hypothesis, in which the baseline measure of MOXO-CPT would differentiate between ADHD and controls participants, the significant differences were found only in attention and hyperactivity scores, there were no differences between the groups in timing scores, impulsivity or measures including RTs. This lack of discriminating was also found in Grossman et al. (2015) study. It is possible that the distractors in the MOXO-CPT are not compatible for adult participants. CPT tests are designed to test the participants alertness tolerance over time, in the absence of external factors that may trigger it (Oken et al., 2006). It may be, that the distractors in the MOXO-CPT are assisting in keeping the participants alert.

According to the load theory of attention (Lavie, 1995; Lavie et al., 2004), higher levels of perceptual load produce more efficient early selection of the task stimuli and reduce distractions (Lavie, 2005; Forster et al., 2014). It is possible, that the distractors in the MOXO-CPT are altering the task from a low to a high perceptual load task, and are assisting the participants to stay alert, and in that manner, the test is not sensitive enough to detect ADHD patients difficulty to stay alert over time while simultaneously reinforcing and consolidating the learning effect.

Going back to our first hypothesis, we were able to significantly reduce hyperactivity measure in MOXO-CPT, by active tDCS. This reduction was exclusive to ADHD patients and to active tDCS condition. This effect was not observed in the control group, and there were no differences between baseline and sham conditions. Hyperactivity in MOXO-CPT is defined as commission responses that are not coded as impulsive (initial response to non-target), which means commission errors due to motor hyper-responsivity, for example, multiple keystrokes in response to a target and random key pressing.

According to an fMRI study by Cubillo et al. (2012) during sustained attention and motor response inhibition tasks, adults with ADHD demonstrated bilateral under-activation of the DLPFC (among other frontal dysfunctions) as well as presumably compensatory hyperactivation in the cerebellum. It is possible that the active tDCS condition modulated the frontal-cerebral network which resulted in reduced hyperactivity, and since hyperactivity is a motoric trait, it is probably less exposed to learning effect, which was observed in all the other assessment criteria of the MOXO-CPT.

Although the hyperactivity measure is unique to the MOXO-CPT, it can be discussed relative to other tDCS studies in which a sample of ADHD participants was included. In line with our results, Soff et al. (2017) applied activities recorder to induce motor assessment during a version of a CPT test, they demonstrated that adolescents with ADHD showed a significant reduction of motor hyperactivity after anodal left DLPFC tDCS (with cathode over the vertex). It is possible that motor hyperactivity is a specific trait, hence tDCS montages as employed here and by Soff et al. (2017) have greater potential to generate a spreading effect which in turn may cause increased activation of the motor network (Sotnikova et al., 2017).

Within this matter, since hyperactivity is the only sub-component that was not influenced by task repetition, it is important to consider the interaction between neuronal activation state (due to enrolling in the task) and the neuronal effect induced by the tDCS itself. The effect of non-invasive brain stimulation techniques is not always linear, it can vary due to task related processes and individual differences (Romei et al., 2016; Silvanto and Cattaneo, 2017; Jospe et al., 2018). Bortoletto et al. (2015) have demonstrated that anodal tDCS of the right motor cortex while performing a task that induced learning (through high neural activation state), hindered performances. Inversely, when task-related activation state was moderate, and the task did not induce learning, enhanced performances were observed.

In relation to the current study, motor hyperactivity trait can be referred as moderate task-related neural activation; CPT tests are intended to measure sustained attention, they are not designed as a pure motor task, thus it is not their only nor their main component. Specifically, the MOXO-CPT is relatively unchallenging, since it intended to discriminate between significantly impaired to average performances. Therefore, according to Bortoletto et al. (2015), during this kind of tasks, the motor cortex is less activated, thus, we were able to detect the significant impairment of ADHD participants. Furthermore, when tDCS was applied, the interaction between the task-related neural state and neural state induced by the tDCS, may result in learning effect, which was exclusive to those who initially exhibit impairment, that is the ADHD patients.

In addition, other tDCS studies with sustained attention tasks (such as Go/NoGo, or CPT versions) in ADHD patient used a total of commission error rate as a measure of impulsivity or inhibitory accuracy. Referring to these measures as an indication to our finding, it is in line with Soltaninejad et al. (2015) and Bandeira et al. (2016) that applied anodal tDCS left DLPFC in children and adolescents with ADHD and demonstrated reduced error rate/increased inhibitory accuracy, respectively (Soltaninejad et al., 2015; Bandeira et al., 2016). In contrast, Cosmo et al. (2015) reported that anodal left DLPFC had no influence on behavioral performances in a Go-NoGo task in a sample of adults with ADHD (Cosmo et al., 2015). We were also not able to demonstrate improvements in the behavioral performances following stimulation, even though there were major methodological differences between the montage, intensity, and duration we employed to what was done by Cosmo et al. (2015). Nevertheless, tDCS for clinical patients is still in its infancy, so more attempts to develop efficient therapeutic interventions are needed.

The limitations of the study are concerning the learning effect in the MOXO-CPT, which leaves some of our hypotheses in place, and the need to replicate this study with a different CPT task. Furthermore, although Soff et al. (2017) demonstrated reduction of hyperactivity a week after the stimulation, since we used offline stimulation (which might reduce the ability to observe the tDCS effects), it is possible that with online stimulation we could overcome or influence beyond the learning effect. In addition, it might be difficult to generalize our results since our participants are ADHD university students, and according to Barkley et al. (2008), ADHD students differ in their advanced coping skills and positive past experiences with school success from non-students ADHD adults. However, they fall behind their non-ADHD university peers; they report more academic problems, poorer studying abilities and organization skills, and difficulty staying motivated (Barkley et al., 2008; DuPaul et al., 2009). It is possible that with non-university ADHD adults we could find greater differences in the MOXO-CPT measures between ADHD subjects and controls.

## Conclusion

What we can learn from this study is that possible repetition effects need to be considered carefully when designing within-subject, multiple tDCS session experiments. It stems from the mere repetition of the cognitive tasks, which are the target function of the experiment, might improve performance and thus concealing potential stimulation effects.

## Author Contributions

This study was part of NJ Ph.D. under the supervision of ML.

## Conflict of Interest Statement

The authors declare that the research was conducted in the absence of any commercial or financial relationships that could be construed as a potential conflict of interest.
